# Socio-Demographic, Health and Lifestyle Factors Influencing Age of Sexual Initiation among Adolescents

**DOI:** 10.3390/ijerph15091851

**Published:** 2018-08-27

**Authors:** Lorraine Burke, Saoirse Nic Gabhainn, Colette Kelly

**Affiliations:** Health Promotion Research Centre, Discipline of Health Promotion, School of Health Sciences, National University of Ireland Galway, H91 TK33 Galway, Ireland; saoirse.nicgabhainn@nuigalway.ie (S.N.G.); colette.kelly@nuigalway.ie (C.K.)

**Keywords:** sexual behaviour, sexual initiation, early sex, adolescents, Ireland

## Abstract

Behavioural and developmental factors mean that adolescents who initiate sexual intercourse early may be at an increased risk of adverse sexual health outcomes at the time of first sex and later in life. In an Irish context, there is insufficient knowledge about the specific correlates of early sexual initiation. This research explores relationships between contextual socio-demographic, health and lifestyle factors and the timing of first sexual intercourse among 15–17-year-olds in Ireland. Multiple regression analysis was carried out in conjunction with Multiple Imputation using data collected through the 2014 Health Behaviour in School-Aged Children Ireland study on a sample of 879 sexually active adolescents. The socio-demographic and lifestyle factors measured were a stronger predictor of age of sexual initiation among girls than boys. Risk behaviour initiation was significantly related to age of sexual initiation for adolescents, while alcohol use/drunkenness and unhealthy food consumption was significant among girls only. Family support and number of male friends were significant predictors for boys only. The study highlights the need for holistic approaches to sexual health promotion and provides a foundation for the development of alternative strategies and policies aimed at reducing negative health, well-being, educational and economic outcomes.

## 1. Introduction

Adolescence is an important life stage involving physical, emotional, social as well as sexual development. Behavioural and developmental factors mean that adolescents who initiate sexual intercourse early may be at an increased risk of adverse sexual health outcomes at the time of first sex and later in life. The initiation of sexual intercourse is an important life event for both females and males in all societies and is an indicator of both physical and psychological development [1]. For many people, sexual initiation is an event that occurs during adolescence [2,3]. While sexual intercourse itself is not necessarily a risk behaviour, it may represent a threat to health and well-being when it occurs at an early age [4]. The timing of sexual initiation, as well as the context and circumstances under which it occurs, can have consequences in the short term and also later in life [1,5].

Sexual activity and the development of sexual relationships are anticipated behaviours of adolescence and should not necessarily be considered deviant. However, all societies have their own attitudes and norms regarding acceptable sexual and reproductive behaviour among adolescents [5]. In many countries the onset of sexual activity among adolescents often does not correspond with the age that is regarded as appropriate within the society and that can result in gaps in the adequate promotion of young people’s sexual health [6]. This highlights the importance of collating up-to-date information on the sexual behaviours and practices of adolescents, in particular important sexual milestones such as first sexual intercourse.

Early sexual initiation has been associated with other risky sexual behaviours such as sexual intercourse under the influence of alcohol or drugs and increased numbers of sexual partners [7,8], as well as experience of forced sex or dating violence [9]. Having sex at an early age has also been linked to a higher risk of experiencing delinquency later in life [10], a greater risk of emotional and behavioural problems or depressive symptoms in adolescence [11,12,13], and lower educational attainment [14,15,16]. Early sexual initiation has also previously been identified as a moderating factor for existing disparities in sexual health and risk behaviours between third level students and their non-student peers [17].

Early sexual behaviour may have different health and behavioural outcomes for males and females. Research has associated early sexual initiation among girls with increased levels of internalising symptoms, although this effect may decline over time affecting adult women less [11,18]. Early initiating girls are less likely to use condoms and other contraceptives at first sex, increasing their risk of pregnancy and Sexually Transmitted Infections (STIs), and are more likely to engage in subsequent sexual risk taking behaviour in adolescence and later in life [19,20,21]. Early sexual activity in females has also been related to higher levels of gynaecological problems [5]. For males, early sex has been associated with an increased likelihood of subsequent risk-taking behaviour, and a higher risk of developing a substance use disorder [22,23]. The current literature suggests that females are more susceptible to experiencing multiple risk factors associated with early sexual initiation than males. While these studies highlight the possible negative outcomes of early sexual initiation, it is also important to note that many early initiators go on to experience positive sexual health outcomes [24,25]. Younger age of sexual initiation in itself may not necessarily be a negative behaviour, rather it is the social, cultural and economic expectations and tasks associated with this life stage that actually render it harmful. Some researchers argue that teenage pregnancy does not in fact constitute a public health issue and that biologically pregnancy at older ages represents a much greater risk [26]. However, other research points to the reality that pregnancy at a young age has the potential to impede educational, career and social achievement [27].

Risk behaviours such as alcohol and substance use have consistently been recognised as correlates of early sexual activity [4,28,29], although an absence of this link has been noted among some populations such as African-Americans [30]. Family factors such as family structure, monitoring, communication and support have also been identified as significant predictors of age of sexual initiation [4,31,32], as have peer factors such as social or perceived norms and composition of close friendship groups [31,33,34,35]. In addition, school level factors have been associated with early sexual initiation. Positive school attachment and achievement are reported as protective influences [4,36].

Sexually activity at young ages has also been associated with a number of other negative circumstantial factors at the time of first sex such as differences in the willingness of partners and reasons for engaging in intercourse as well as issues of consent [37,38]. Wellings et al. 2001, composed a measure of sexual competence pertaining to the circumstances of early heterosexual experience. This measure was composed of variables assessing willingness; regret, autonomy and contraception use at the time of first intercourse [39]. Using this definition of sexual competence, Wellings et al. reported that lack of sexual competence increased with declining age of sexual initiation and prevalence of sexual non-competence was far higher among both men and women who left school before age 16 years without qualifications [39].

Previous international research has linked social class with sexual initiation among adolescents [36], and this has also been identified in an Irish context [40]. Hierarchical models of influence such as Bronfenbrenner’s ecological systems theory or Dahlgren and Whitehead’s social determinants of health point to the many layers of influences and interactions between different environments that mould and shape human development and behaviours [41,42]. These theoretical perspectives suggest that no behaviour occurs in isolation and that behaviours are heavily influenced by the context and environment in which they occur, as well as wider social and cultural settings, and so it is necessary to explore multiple factors when investigating adolescent sexual behaviour.

In the context of Ireland, sexual health data are relatively scarce. A retrospective study of sexual initiation in Ireland found that from the 1940’s to the 1980’s the median age of first sexual intercourse decreased from 22/23 years to 17 years [2] a pattern identified in multiple developed countries [43]. The Health Behaviour in School-Aged Children (HBSC) Ireland study collected the first nationally representative and internationally comparable data on adolescent sexual behaviour in Ireland in 2010. Findings from this study reported that 25.7% of boys and 21.2% of girls aged 15–18 years had experience of sexual intercourse and also reported levels of contraceptive use at last intercourse, with very early sexual initiation (<14 years) reported by 22.8% of sexually initiated boys and 13.4% of sexually initiated girls [35]. The most recent HBSC international study reported that of 15 year-old participants, 14% of girls and 21% of boys from Ireland reported engaging in sexual intercourse, compared to the HBSC average of 17% of girls and 24% of boys from the participating 40 countries [3].

The main source of sexuality education for adolescents in Ireland is through the school curriculum in the form of a Relationships and Sexuality Education (RSE) programme. The content of RSE provided by schools is not mandated, is often inconsistent and is generally guided by a school’s individual policy which is underpinned in the majority of cases by a Catholic religious ethos [44]. The need for improvements in sexual education and sexual health promotion in Ireland is highlighted by the increasing trends in STI acquisition with notifications increasing by 13% in 2016/2017 [45]. The burden of STI’s is seen among 15–24 years with young people accounting for 50% of all chlamydia cases reported [45]. While teenage pregnancy has declined in recent years dropping from 17.8 per 1000 live births in 2007 to 6.9 per 1000 in 2017 [46], 66% of women and 31% of men aged 18–25 years reported crisis circumstances of pregnancy in the most recent nationally collected data [47]. In the context of Ireland there is insufficient knowledge about the specific correlates of early sexual initiation and so little is known as to whether sexual health promotion approaches used in other countries would be relevant in an Irish setting. The aim of this study is to explore socio-demographic, lifestyle and behavioural factors associated with early timing of first sexual intercourse specific to adolescents in an Irish context. Identifying the most important predictive and protective correlates of early sexual initiation can guide decisions on choosing routes for interventions and initiatives aimed at reducing the negative outcomes of early sex.

## 2. Materials and Methods

### 2.1. Participants

A total of 230 primary and post primary schools and 13,611 children took part in the 2014 Health Behaviour in School-Aged Children (HBSC) Ireland survey. The sample for this study included 3928 participants aged 15 to 17 years, 879 of whom reported ever having sexual intercourse. The sample used in this study included only sexually active participants and the analytical sample consisted of the 436 boys and 443 girls, aged 15 to 17 years, who reported ever having sex. Prevalence of the socio-demographic characteristics of the study sample and the non-sexually active sample are reported in Table 1. Significant differences were observed in some of the socio-demographic characteristics of the sexually active and non-sexually active samples.

All subjects gave their informed consent for inclusion before they participated in the study. The study was conducted in accordance with the Declaration of Helsinki, and the protocol was approved by the Ethics Committee of the National University of Ireland Galway (Project identification code: 13/NOV/14).

### 2.2. Procedure

The HBSC study is a World Health Organisation (WHO) cross-national research project that collects data from students aged 11, 13 and 15-year-olds every four years across 44 countries [48]. Participating children were asked to answer questions on socio-demographic factors, lifestyle factors, as well as health behaviours and outcomes using a self-completion internationally standardised questionnaire. Questionnaires were administered in the classrooms of primary and post-primary schools comprising a nationally representative sample of school children aged 9–17 years across eight geographical regions in the Republic of Ireland. Schools were randomly selected and only children who assented and whose parents provided informed consent (either active or passive) were eligible to take part. Ethical approval was granted by the Human Research Ethics Committee of the National University of Ireland Galway, Ireland. In accordance with the international HBSC protocol, sexual behaviour items were asked only of students aged 15 years and older as the majority of adolescents younger than this are unlikely to have engaged in sexual intercourse and there is a possibility that schools and parents may deem such questions to be too sensitive to ask of younger participants.

### 2.3. Measures

All questionnaire items included in the analysis were designed or adapted by the international HBSC study network and were subjected to piloting and pre-testing protocols [40]. Individual socio-demographic and lifestyle measures were identified from the HBSC 2014 survey data relating to the following nine themes; (1) risk behaviours, (2) school, (3) health behaviours, (4) environment, (5) peers, (6) self-perception, (7) family, (8) health status and (9) environment. A series of exploratory factor analyses were carried out to investigate the possibility of reducing the number of variables within each theme of interest to a smaller number of combined factors. An individual principal component analysis (PCA) was conducted on each set of items for chosen themes using orthogonal rotation (Varimax). The overall Kaiser–Meyer–Olkin (KMO) for each set of theme items verified the sampling adequacy for the analyses with all KMO values above the acceptable limit of 0.5 [49]. All KMO measures for individual items were also above the threshold of 0.5. Bartlett’s test of sphericity was statistically significant for all sets of theme items (*p* < 0.001), indicating that correlations between items were large enough for PCA. These individual thematic factor analyses were used as a guide for the creation of scales in the multiple imputation (MI) data set, as the process is not compatible with MI. Only factors with three or more loading variables were used as templates for scales [50]. Important items of interest such as; school type, urban/rural status, disability/chronic illness status, self-reported health, number of evenings spent with friends, days spent with friends after school, number of close male/female friends and living with father in main home, that did not successfully load onto factors were included in subsequent analyses as individual variables. Scales were created by computing the sum of the component question items and all variables were coded in the same direction ahead of the scale creation process. Reliability analysis was carried out on each variable scale created in the data set with all scales resulting in acceptable Cronbach’s alpha values of greater than 0.6. Question wordings, final versions of measures and their labels are reported in Supplementary Material Table S1.

### 2.4. Statistical Analysis

While the amount of missing data on any independent variable was small (<10% missing), multiple imputation (MI) was implemented to avoid dropping participants from the analytical sample due to missing responses on some variables. All independent, predictor and dependent variables were included in the imputation model. The potential confounding variables of gender and social class were included in the imputation model only as predictor variables and were not imputed. The ‘Automatic’ imputation method was chosen on SPSS which scans the data to identify if missing responses show a monotone pattern. Pooled estimates of complete cases were obtained based on ten imputations [51]. Due to the fact it was an MI data set, variables in the analysis were standardised in order to allow interpretation of β coefficient outputs from multiple regression analysis. Model summary estimates were calculated based on Rubin’s Rules [51]. All analyses were conducted using SPSS 23 (IBM Corp, Armonk, NY, USA).

Initial multiple linear regression analyses were carried out separately for each of the identified themes. Measures that emerged as significant predictors of age of sexual initiation (ASI) within individual regressions were isolated and the predictive power R^2^ of their over-arching themes were ranked. These rankings were used to form the basis of a hierarchical multiple linear regression containing all significant coefficients from each theme. Those themes with the highest R^2^ values had their corresponding significant factors entered first, with each step of the regression represented by the addition of another theme. Hierarchical multiple regressions were then conducted with ASI as the dependent variable. Any independent variables not significantly associated with ASI were excluded from the hierarchical analyses. Different measures and themes emerged as significant predictors among boys and girls in the individual regressions. All regressions were carried out separately for boys and girls. All relevant assumptions were tested prior to conducting all multiple regressions analyses [52].

## 3. Results

### 3.1. Descriptive Statistics

Bivariate associations between study variables are reported in Supplementary Material Table S2, for boys and girls. A Pearson’s product-moment correlation was run to assess the relationship between continuous variables. Phi coefficients were calculated to assess the relationship between dichotomous variables and Point-Biserial correlations were calculated to assess the relationship between dichotomous versus continuous variables.

### 3.2. Predictors of Early Sexual Initiation among Boys

A seven-stage hierarchical multiple regression analysis was conducted with age of sexual initiation as the dependent variable. Social class was entered at Step 1 to control for social effects and class influences. Peer, family, risk behaviour, school, life perception, environment, health status and health behaviour measures were entered in subsequent steps. Table 2 demonstrates that all models were statistically significant, including the overall model (Model 7). The addition of peer factors at Step 2 provided the largest contribution to the model uniquely explaining 4.4% of the variation in ASI, with number of male friends also a significant coefficient in the regression model. Other significant coefficients emerging from the regression model were family support and risk behaviour initiation.

### 3.3. Predictors of Early Sexual Initiation among Girls

A nine-stage hierarchical multiple regression analysis was conducted with age of sexual initiation as the dependent variable. Social class was entered at Step 1 to control for social effects and class influences. Risk behaviour, school, health behaviour, health status, peer, family, environment and life perception measures were entered in subsequent steps. Table 3 demonstrates that all models were statistically significant, with the exception of Model 1 which consisted of social class only. The inclusion of risk behaviour measures at Step 2 provided the largest contribution to the model accounting for 19.8% of the variation in ASI, with alcohol use/drunkenness and risk behaviour initiation also emerging as significant coefficients in the regression model. Unhealthy food consumption also emerged as a significant coefficient.

Overall, the sociodemographic, lifestyle and behavioural factors included in the analysis were stronger predictors of age of sexual initiation among sexually active girls than boys. Together the included predictors accounted for 23.2% of variation in ASI for sexually active girls aged 15 to 17 years, and 10.9% of the variation in ASI was explained for boys.

## 4. Discussion

The study found that initiation of risk behaviours such as smoking, alcohol use, drunkenness or cannabis at younger ages was predictive of early sexual initiation among girls and boys. Sexual risk behaviours and smoking, alcohol and substance use have consistently been linked in the literature and are often considered to occur in clusters thus the finding that initiation of these behaviours may be related in an Irish context is no surprise [4,28]. In contrast, girls who reported lower levels of alcohol consumption or experience of being drunk were also more likely to have had sex at younger ages. This finding is in conflict with previous research reporting higher frequency of alcohol use at age 16 years by girls who experienced early first sexual intercourse [29]. While this may appear to be a positive outcome, the reason of this association is not known and may even be linked to a negative first intercourse experience involving substance or alcohol use which has been associated with higher levels of regret around first sexual encounter, and may in turn deter girls from subsequent alcohol or drug related behaviours [53]. An alternative explanation for this association may be relationship status. Studies have linked romantic relationships with early sexual experience [54,55] and being in a romantic relationship could in turn alter the social behaviour of adolescents and potentially reduce their involvement in risk behaviour. Sociological research has also posited that adolescent romantic relationships may lead to partners developing a level of attachment, and this trait has been linked to more positive social behaviours, as well as decreases in drug and alcohol use among other behaviours [56,57]. Of the risk behaviour measures included, age of risk behaviour initiation was a stronger predictor of ASI among girls than alcohol use or drunkenness per se.

Among sexually active girls, a higher consumption of unhealthy food was a predictor of younger ages of first sexual intercourse. As mentioned previously, risk behaviours such as substance use have been known to cluster with early sexual activity [28], alcohol use has also been linked with other negative health behaviours such as physical inactivity and low fruit and vegetable intake among adolescents [58]. However, a systematic review by Wiefferink et al., in 2006 failed to find proof of clustering of health-enhancing behaviours such as healthy nutrition and safe sex, although they also did not find any evidence that the behaviours were not clustered [59]. Unhealthy eating habits and early sexual initiation are both health compromising behaviours and there is the possibility that these behaviours may represent some level of risk behaviour clustering unique to adolescent girls. Although this warrants further investigation, it may reinforce the importance that health promoting interventions tackle multiple health risk behaviours using an integrated approach [58,59,60]. From another perspective, childhood sexual abuse has been linked to obesity and so there is a possibility that if sexual intercourse was a negative experience it may have repercussions for dietary behaviour [61]. Such speculation requires further investigation.

Among boys, those who had fewer close male friends were more likely to first have sex at younger ages than those with more friends. The mechanism of this is unclear but may be related to social norms or perceptions as intention to engage in, as well as initiation of sexual intercourse is influenced by perceived levels of peer sexual activity [33,34,35]. Having a larger peer network may lead to a more accurate assessment of peer sexual initiation and hence protect against early engagement in sexual intercourse.

The results also identified boys who have a supportive family are less likely to have reported first sexual intercourse at younger ages. This corresponds with existing evidence citing positive family factors as protective against negative health behaviours, and risky sexual behaviour [4,32,62]. However, more research is warranted in order to decipher why this may be a stronger protective factor for boys than for girls.

While some lifestyle and health behaviour factors are common to those adolescents who engage in early sexual initiation, there are different factors that are important for boys and girls separately. This study has confirmed previous research findings around the interlinking nature of risk behaviours and their initiation and the importance of family level factors [4,32,63]. It also suggests that the relationship between early sex and subsequent alcohol use among adolescent girls may be more nuanced than previously thought. The study has also identified the possible relationship between early sex and eating habits of girls. Aside from risk behaviour initiation, behavioural characteristics (alcohol/drunkenness, dietary habits) at the individual level appeared to be significant influencers of ASI among girls, whereas more microsystem level factors such as peers and family were significant for boys.

The findings indicate the importance of providing adolescents with adequate sex education at younger ages, earlier even than through the post-primary school system, in order to provide them with the knowledge, skills and understanding to effectively navigate sexual situations and make positive health decisions as they develop. The findings also emphasise the need for a more a holistic approach to positive sexual health and well-being, including targeting a number of interrelated behaviours in conjunction with sexual health—as opposed to basing interventions and education around sexual health in isolation. Another approach that could be taken in order to alleviate the negative outcomes of early sexual initiation could be to promote or increase behaviours that are generally protective in nature. For instance, promoting good family support or increasing one’s network of friends and supports. As this study and others have highlighted, different factors influence boys and girls in relation to early sexual initiation, it may be beneficial to tailor programmes to suit specific genders providing specialised approaches in order to overcome gender specific barriers to positive sexual health. This could be particularly relevant in Ireland where a third of post-primary schools are single-sex [64,65]. Ireland has a particularly problematic relationship with alcohol and many formal and informal life events in Ireland often occur in the presence of alcohol [66]. This study has linked initiation of other risk behaviours with early sexual intercourse and so there is every possibility that alcohol consumption may play a part in this life event for adolescents in Ireland. This provides rationale further targeting a number of risk behaviours together in order to improve well-being outcomes of adolescents. Though this study sample included only adolescents in school there is also potential to use these findings to inform sexual health and behaviour initiatives and interventions in an out-of-school setting such as local youth centres.

Overall, reliable information on the patterns of sexual initiation timing, as well as factors influencing this timing, can support more effective education and intervention design targeted at stages where it can have the best possible impact among adolescents. This could equip adolescents, particularly those who have sex at young ages, with the skills and competence they need to successfully navigate sexual situations and events socially, emotionally and physically.

### Limitations

This population-based study has provided important data on the predictors of early sexual initiation of adolescents in Ireland. However, the study is not without its limitations. For instance, while an explanation is provided for sexual intercourse it does not specify an anatomical definition. The study also does not include a measure of sexual consent which is an important factor in the circumstances of sexual intercourse, particularly among adolescents, and so capturing non-consensual sex is beyond the scope of this research. The study also lacks a measure of romantic relationship status which could potentially act as a mediator or predictor of age of sexual initiation or associated behaviours. Inclusion of measures of romantic relationship status, willingness, autonomy and consent would greatly broaden the scope of future research in this area and improve understanding around the specific circumstances of first sexual intercourse.

While this study focuses on a narrow age range of 15–17 years, developmentally there may be considerable difference between the age groups. However, this study has not explored differences in experience of sexual initiation and its’ predictors by age. As this study explores the explanatory factors associated with age of first sex among a narrow, and relatively young age sample there may be less variability in these factors than a sample more heterogeneous for age. Another limitation of the study sample is the fact that data is collected solely from adolescents who are attending school. While this provides an easily accessible and relatively large sample, it fails to take into account the experiences of out-of-school youth, who may even be at a greater risk of negative lifestyle and health behaviours due to social and environmental factors.

As this study focuses on adolescents who are already sexually active, and in particular those who had sex early, it is important to note that the findings may not be relevant to all adolescents. Factors that were found to be significant predictors of early sexual activity may not necessarily be influencing factors for age of sexual initiation for the majority of adolescents who do not engage in early sexual intercourse. However, as early sexual initiation has previously been identified as more risky in terms of health and well-being outcomes [4,5] focusing on this minority is warranted in order to improve the sexual health outcomes of those adolescents most at risk. In this study we have observed some differences in the sexually active and non-sexually active participants. In particular, there are significantly more younger adolescents, rural adolescents, higher social class girls, and girls attending single-sex schools in the non-sexually active population (see Table 1).

The cross-sectional nature of the data also prevents causality from being inferred between age of sexual initiation and significant predictors. In order to further our understanding of the mechanisms underlying the patterns reported here qualitative exploration of the possible causes or explanations for these associations from the point of view of adolescents themselves is warranted.

## 5. Conclusions

This study greatly enhances the previously limited research available around adolescent sexual behaviour at a time where this particular topic is very much to the fore in the Irish context. As a result of a recent abortion referendum, as well as public attention focusing on the issue of consent, the current political climate in Ireland is very much concerned with positive sexual health promotion and its many facets. In fact, the Minister for Education has recently mandated a review and update of Ireland’s 20 years old RSE curriculum. This may provide an ideal opportunity to present these findings in order to update Irish sex education in an evidence-based manner for children and adolescents in Ireland.

## Figures and Tables

**Table 1:** Prevalence of socio-demographics among the sexually active and non-sexually initiated samples, by gender.

	Sexually Active		Non-Sexually Active	
	Boys (n = 436)	Girls (n = 443)	Boys (n = 954)	Girls (n = 1627)
Age				
15 years	138 ^a^ (31.7%)	120 ^a^ (27.1%)	392 ^b^ (41.1%)	731 ^b^ (44.9%)
16 years	173 ^a^ (39.7%)	172 ^a^ (38.8%)	357 ^a^ (37.4%)	618 ^a^ (38.0%)
17 years	125 ^a^ (28.7%)	151 ^a^ (34.1%)	205 ^b^ (21.5%)	278 ^b^ (17.1%)
Social class				
High (1–2)	168 ^a^ (49.9%)	180 ^a^ (48.8%)	468 ^a^ (55.0%)	796 ^b^ (54.9%)
Mid (3–4)	122 ^a^ (36.2%)	138 ^a^ (37.4%)	295 ^a^ (34.7%)	517 ^a^ (35.7%)
Low (5–6)	47 ^a^ (13.9%)	51 ^a^ (13.8%)	88 ^a^ (10.3%)	137 ^b^ (9.4%)
Urban/rural status				
Urban	206 ^a^ (48.6%)	247 ^a^ (56.3%)	354 ^b^ (37.3%)	717 ^b^ (44.4%)
Rural	218 ^a^ (51.4%)	192 ^a^ (43.7%)	594 ^b^ (62.7%)	899 ^b^ (55.6%)
School type				
Single gender	115 ^a^ (26.4%)	207 ^a^ (46.7%)	220 ^a^ (23.1%)	878 ^b^ (54.0%)
Mixed gender	321 ^a^ (73.6%)	236 ^a^ (53.3%)	734 ^a^ (76.9%)	749 ^b^ (46.0%)

Note: Each superscript letter denotes a subset of ever had sex categories whose column proportions do not differ significantly from each other at the 0.05 level.

**Table 2:** Hierarchical linear regression model results, predictors of age of sexual initiation among boys.

	Model 1	Model 2	Model 3	Model 4	Model 5	Model 6	Model 7
	β	β	β	β	β	β	β
Step 1: Control							
Social class	0.158 **	0.101	0.086	0.083	0.079	0.078	0.071
Step 2: Peers							
Peer support		0.138 *	0.071	0.071	0.066	0.060	0.060
Number of close male friends		0.198 **	0.176 **	0.175 **	0.18 **	0.175 **	0.165 *
Step 3: Family							
Family support			0.165 **	0.144 *	0.136 *	0.135 *	0.135
Step 4: Risk behaviours							
Risk behaviours initiation				−0.153 **	−0.136 *	−0.129 *	−0.129 *
Step 5: School							
Relationship with teachers and school					−0.065	−0.048	−0.043
Step 6: Life perception							
Happiness and satisfaction with life						0.050	0.033
Step 7: Environment							
Positive neighbourhood environment							−0.052
R^2^	0.022	0.065	0.083	0.103	0.106	0.107	0.109
∆R^2^	0.022	0.044	0.018	0.020	0.003	0.002	0.002
Adjusted R^2^	0.019	0.059	0.074	0.092	0.093	0.093	0.093
F	9.52 **	10.05 ***	9.74 ***	9.84 ***	8.46 ***	7.35 ***	6.53 ***

* *p* ≤ 0.05; ** *p* ≤ 0.01; *** *p* ≤ 0.001.

**Table 3:** Hierarchical linear regression model results, predictors of age of sexual initiation among girls.

	Model 1	Model 2	Model 3	Model 4	Model 5	Model 6	Model 7	Model 8	Model 9
	β	β	β	β	β	β	β	β	β
Step 1: Control									
Social class	0.046	−0.005	−0.003	−0.026	−0.028	−0.026	−0.025	−0.026	−0.024
Step 2: Risk behaviours									
Alcohol use or drunkenness		0.242 ***	0.252 ***	0.245 ***	0.245 ***	0.242 ***	0.242 ***	0.242 ***	0.241 ***
Risk behaviours initiation		−0.43 ***	−0.401 ***	−0.386 ***	−0.379 ***	−0.371 ***	−0.37 ***	−0.369 ***	−0.372 ***
Step 3: School									
Relationship with teachers and school			−0.089	−0.079	−0.064	−0.063	−0.062	−0.059	−0.068
Step 4: Health behaviours									
Unhealthy food consumption				−0.098 *	−0.094 *	−0.092 *	−0.092 *	−0.09 *	−0.086 *
Step 5: Health status									
Physical & psychological symptoms					0.056	0.05	0.048	0.043	0.072
Step 6: Peers									
Number of close female friends						0.064	0.064	0.061	0.067
Number of close male friends						−0.037	−0.038	−0.039	−0.040
Step 7: Family									
Family communication							−0.008	−0.006	−0.020
Step 8: Environment									
Positive neighbourhood environment								−0.019	−0.028
Step 9: Life perception									
Happiness and satisfaction with life									−0.074
R^2^	0.003	0.198	0.208	0.219	0.224	0.227	0.228	0.228	0.232
∆R^2^	0.003	0.195	0.010	0.012	0.004	0.004	0.000	0.000	0.004
Adjusted R^2^	0.001	0.192	0.200	0.211	0.213	0.213	0.210	0.210	0.213
F	1.23	36.08 ***	28.69 ***	24.61 ***	20.94 ***	15.97 ***	12.75 ***	12.75 ***	11.85 ***

* *p* ≤ 0.05; ** *p* ≤ 0.01; *** *p* ≤ 0.001.

## Supplementary Materials

The following are available online at http://www.mdpi.com/1660-4601/15/9/1851/s1. Table S1: Item wordings, measures and labels, by theme, Table S2: Bivariate associations between study variables, for boys (bottom left), and girls (top right).
