# Supplementary material for: Socio-Demographic, Health and Lifestyle Factors Influencing Age of Sexual Initiation among Adolescents
Source: Int J Environ Res Public Health. 2018 Aug 27;15(9):1851. doi: 10.3390/ijerph15091851 (PMC6163828; doi:10.3390/ijerph15091851)
Supplement: Supplementary file 1 [file ijerph-15-01851-s001.zip › ijerph-334723-supplementary.docx]

**Supplementary Table S1.** Item wordings, measures and labels, by theme.

| **Label** | **Themes and item measures** | **Direction of measures** |
| --- | --- | --- |
|  | **Dependent** |  |
| ASI | *‘How old were you when you had sexual intercourse for the first time?’ | 11 to ≥ 17 years |
|  | **Control** |  |
| SC | *Social Class | Unskilled to professional |
|  | **Risk behaviours** |  |
| TC | *Tobacco or cannabis use - (Scale) | None to increasing use |
|  | ‘How frequently have you smoked cigarettes during the last 30 days?’ |  |
|  | ‘How often do you smoke tobacco at present? |  |
|  | ‘On how many days (if any) have you smoked cigarettes? .....In your lifetime’ |  |
|  | ‘Have you ever taken cannabis (hashish, grass, pot)? …..In your lifetime’/in the last 30 days’ |  |
| AD | *Alcohol use or drunkenness - (Scale) | None to increasing use |
|  | ‘Have you ever had so much alcohol that you were really drunk? ..... In your lifetime’/in the last 30 days’ |  |
|  | ‘On how many days (if any) have you drunk alcohol? .... In your lifetime’/in the last 30 days’ |  |
| RBI | *Risk behaviours initiation - (Scale) | 11 or younger to never |
|  | ‘At what age did you first do the following things? |  |
|  | Drink alcohol (more than a small amount)/get drunk/ Smoke a cigarette (more than a puff)/take cannabis |  |
|  | **School** |  |
| RTS | *Relationship with teachers and school - (Scale) | Positive to negative |
|  | ‘I feel a lot of trust in my teachers’ |  |
|  | ‘I feel that my teachers accept me as I am’ |  |
|  | ‘I feel that my teachers care about me as a person’ |  |
|  | ‘In your opinion, what does your class teacher(s) think about your school performance compared to your classmates?’ |  |
|  | ‘How do you feel about school at present?’ |  |
|  | ‘I am encouraged to express my own views in my class(es)’ |  |
| RS | *Student support - (Scale) | Positive to negative |
|  | ‘The students in my class(es) enjoy being together’ |  |
|  | ‘Most of the students in my class(es) are kind and helpful’ |  |
|  | ‘Other students accept me as I am’ |  |
|  | ‘Students get involved in organising school events’ |  |
| SE | *School environment - rules, safety, student participation - (Scale) | Positive to negative |
|  | ‘In our school the students take part in making the rules’ |  |
|  | ‘The rules in this school are fair’ |  |
|  | ‘I feel safe at this school’ |  |
| ST | School type | Unmixed/Mixed gender |
|  | **Environment** |  |
| NH | *Neighbourhood - good, safe, helpful neighbours - (Scale) | Positive to negative |
|  | ‘Do you think that the area in which you live is a good place to live?’ |  |
|  | ‘Generally speaking, I feel safe in the area where I live…..’ |  |
|  | ‘I could ask for help or a favour from neighbours’ |  |
| URL | *‘Where do you live?’ | Urban/rural |
|  | **Health status** |  |
| PPS | *Physical and psychological symptoms - (Scale) | Rarely to often |
|  | ‘In the last 6 months: how often have you had the following….? Headache/ stomach-ache/back ache/ feeling low/ irritability or bad temper/ feeling nervous/ difficulties in getting to sleep/feeling dizzy’ |  |
| DCI | *‘Do you have a long-term illness, disability, or medical condition (like diabetes, asthma, allergy or cerebral palsy) that has been diagnosed by a doctor?’ | Disability or chronic illness/not |
| SRH | *‘Would you say your health is……?’ | Poor to excellent |
|  | **Health behaviours** |  |
| UFC | *Unhealthy food consumption - (Scale) | Never to daily+ |
|  | ‘How many times a week do you usually eat or drink .... ? Crisps, coke or other soft drinks that contain sugar, chips/fried potatoes, sweets (candy or chocolate), diet coke or diet soft drinks’ |  |
| PA | *Physical activity and exercise - (Scale) | Less to more |
|  | ‘Over the past 7 days, on how many days were you physically active for a total of at least 60 minutes per day?’ |  |
|  | ‘How often do you usually exercise in free time so much so that you get out of breath or sweat?’ |  |
|  | ‘How many hours do you usually exercise in free time so much so that you get out of breath or sweat?’ |  |
| HFC | *Healthy food consumption - (Scale) | Never to daily+ |
|  | *‘How many times a week do you usually eat or drink .... ? Vegetables/fruits/fish’ |  |
|  | **Peers** |  |
| FS | *Peer support - (Scale) | Positive to negative |
|  | ‘I have friends with whom I can share my joys and sorrows’ |  |
|  | ‘I can talk about my problems with my friends’ |  |
|  | ‘I can count on friends when things go wrong’ |  |
|  | ‘My friends really try to help me’ |  |
| FC | *Comfortable talking to friends and being yourself - (Scale) | Positive to negative |
|  | ‘How easy is it for you to talk to the following persons about things that really bother you? |  |
|  | Best friend/ friend of the same sex/ friend of the opposite sex’ |  |
|  | ‘Do you feel comfortable being yourself while with friends?’ |  |
| EF | *‘How many evenings per week do you usually spend out with your friends?’ | 0 - 7 evenings |
| DF | *‘How many days a week do you usually spend time with friends right after school?’ | 0 - 5 days |
| MF/FF | *‘At present, how many close male / female friends do you have?’ | None to 3 or more |
|  | **Family** |  |
| FAMS | *Family support - (Scale) | Negative to positive |
|  | ‘My family is willing to help me make decisions’ |  |
|  | ‘My family really tries to help me’ |  |
|  | ‘I get the emotional support I need from my family’ |  |
|  | ‘I can talk about my problems with my family’ |  |
| FAMC | *Family clear communication - (Scale) | Positive to negative |
|  | ‘In my family 0.0.0.I think the important things are talked about/ when I someone listens when I/when there is a misunderstanding we talk it over until it's clear/ we ask questions when we don't understand each other’ |  |
|  | ‘How easy is it for you to talk to the following persons about things that really bother you? Mother’ |  |
| FIH | *‘Please answer this first question for the home where you live all or most of the time and tick the people who live there…Father’ | Father present/not |
|  | **Life perception** |  |
| HSL | *Happiness and satisfaction with life - (Scale) | Negative to positive |
|  | ‘In general how do you feel about your life at present?’ |  |
|  | ‘The top of the ladder ‘10’ is the best possible life for you and the bottom ‘0’ is the worst possible life for you0. In general, where on the ladder do you feel you stand at the moment?’ |  |
|  | ‘Thinking about the last week…0.0.Have you been happy with the way you are?’ |  |
|  | **Filter** |  |
|  | ‘Have you ever had sexual intercourse? (Sometimes this is called “making love”, “having sex” or “going all the way”)’ |  |
|  | ‘Are you a boy or a girl?’ |  |

**Table S2.** Bivariate associations between study variables, for boys (bottom left), and girls (top right).

|  | **ASI** | **SC** | **TC** | **AD** | **RBI** | **RTS** | **RS** | **SE** | **ST** | **NH** | **URL** | **PPS** | **DCI** | **SRH** | **UFC** | **PA** | **HFC** | **FS** | **FC** | **EF** | **DF** | **FF** | **MF** | **FAMS** | **FAMC** | **FIH** | **HSL** |
| --- | --- | --- | --- | --- | --- | --- | --- | --- | --- | --- | --- | --- | --- | --- | --- | --- | --- | --- | --- | --- | --- | --- | --- | --- | --- | --- | --- |
| ASI |  | 0.05 | -0.20** | 0.08 | -0.38** | -00.21** | -00.08 | -00.15** | -0.11* | -0.15** | 0.04 | 0.20** | -0.03 | 0.15** | -0.21** | 0.01 | 0.14** | -0.03 | -0.03 | -0.11* | -0.05 | 0.08 | -0.08 | 0.10* | -0.13** | 0.08 | 0.07 |
| SC | 0.15** |  | -0.08 | 0.03 | -0.11* | -0.02 | -0.11* | -0.08 | -0.08 | -0.07 | -0.11* | 0.09 | -0.08 | 0.03 | -0.25** | 0.08 | 0.24** | 0.06 | -0.12* | -0.08 | -0.06 | 0.02 | 0.05 | -0.01 | 0.02 | 0.16** | 0.06 |
| TC | -0.13** | 0.01 |  | 0.51** | 0.64** | 0.36** | 0.22** | 0.30** | 0.16** | 0.18** | -0.05 | -0.29** | 0.00 | -0.28** | 0.16** | -0.12* | -0.11* | -0.04 | 0.08 | 0.17** | 0.13** | -0.07 | 0.19** | -0.17** | 0.25** | -0.09 | -0.29** |
| AD | -0.05 | 0.12* | 0.53** |  | 0.46** | 0.23** | 0.12* | 0.19** | 0.05 | 0.10* | -0.07 | -0.15** | -0.04 | -0.05 | 0.02 | -0.06 | -0.04 | 0.05 | -0.02 | 0.18** | 0.11* | 0.10 | 0.15** | -0.05 | 0.16** | 0.01 | -0.17** |
| RBI | -0.17** | -0.04 | 0.57** | 0.45** |  | 0.37** | 0.23** | 0.32** | 0.15** | 0.22** | -0.06 | -0.26** | 0.00 | -0.20** | 0.18** | -0.09 | -0.17** | -0.04 | 0.10 | 0.23** | 0.12* | -0.03 | 0.17** | -0.15** | 0.25** | -0.04 | -0.24** |
| RTS | -0.13* | -0.08 | 0.25** | 0.20** | 0.31** |  | 0.51** | 0.56** | 0.11* | 0.32** | -0.06 | -0.35** | 0.02 | -0.21** | 0.17** | -0.14** | -0.12* | 0.00 | 0.13** | 0.11* | 0.04 | -0.04 | 0.07 | -0.17** | 0.36** | -0.08 | -0.37** |
| RS | 0.02 | -0.04 | 0.10* | 0.13* | 0.15** | 0.38** |  | 0.45** | 0.20** | 0.28** | 0.02 | -0.33** | 0.05 | -0.12** | 0.13** | -0.07 | -0.11* | -0.04 | 0.19** | 0.04 | 0.02 | -0.10* | -0.03 | -0.09 | 0.28** | -0.08 | -0.31** |
| SE | -0.08 | 0.03 | 0.26** | 0.22** | 0.33** | 0.48** | 0.33** |  | 0.05 | 0.27** | -0.04 | -0.28** | 0.07 | -0.15** | 0.17** | -0.05 | -0.15** | 0.10* | 0.02 | 0.12* | 0.11* | 0.06 | 0.03 | -0.07 | 0.20** | -0.03 | -0.24** |
| ST | 0.00 | -0.11* | 0.01 | 0.00 | 0.06 | 0.07 | -0.02 | -0.03 |  | -0.01 | -0.12* | 0.04 | 0.00 | -0.05 | -0.03 | -0.07 | -0.14** | -0.04 | -0.01 | 0.08 | 0.09 | -0.06 | 0.06 | -0.08 | 0.08 | 0.02 | 0.03 |
| NH | -0.15** | -0.20** | 0.14** | 0.10 | 0.12* | 0.23** | 0.22** | 0.16** | 0.06 |  | 0.16** | -0.40** | 0.04 | -0.19** | 0.18** | -0.10* | -0.09 | -0.06 | 0.15** | 0.07 | 0.03 | -0.17** | -0.06 | -0.16** | 0.30** | -0.07 | -0.38** |
| URL | 0.03 | -0.13** | 0.07 | -0.10* | -0.01 | -0.09 | 0.07 | -0.10* | -0.04 | 0.20** |  | 0.01 | 0.04 | -0.01 | 0.10* | 0.01 | -0.04 | 0.01 | -0.02 | 0.20** | 0.11* | -0.04 | -0.01 | 0.05 | -0.05 | -0.10* | -0.02 |
| PPS | 0.12* | 0.11* | -0.18** | -0.18** | -0.25** | -0.24** | 0.19** | -0.19** | 0.07 | -0.23** | -0.03 |  | -0.13** | 0.31** | -0.15** | 0.09 | 0.04 | 0.06 | -0.23** | -0.01 | -0.01 | 0.15** | -0.04 | 0.20** | -0.37** | 0.08 | 0.58** |
| DCI | -0.06 | -0.04 | 0.07 | 0.02 | -0.01 | 0.00 | 0.07 | 0.02 | 0.04 | 0.00 | 0.01 | -0.12* |  | -0.12** | -0.02 | 0.03 | 0.03 | -0.03 | 0.08 | -0.01 | 0.04 | -0.04 | 0.00 | -0.05 | 0.03 | -0.08 | -0.08 |
| SRH | 0.05 | 0.08 | -0.17** | -0.11* | -0.18** | -0.25** | -0.17** | -0.12* | -0.03 | -0.19** | -0.02 | 0.27** | -0.07 |  | -0.14** | 0.17** | 0.13** | 0.05 | -0.15** | -0.08 | -0.04 | 0.02 | -0.04 | 0.15** | -0.24** | 0.13** | 0.33** |
| UFC | -0.05 | -0.10 | 0.13* | 0.10 | 0.16** | 0.12* | 0.01 | 0.07 | -0.01 | 0.12* | -0.08 | -0.12* | 0.07 | -0.09 |  | -0.18** | -0.31** | -0.07 | 0.06 | 0.20** | 0.13* | -0.05 | 0.05 | -0.08 | 0.07 | -0.09 | -0.06 |
| PA | 0.04 | 0.03 | -0.19** | -0.03 | -0.13* | -0.14** | -0.03 | -0.09 | 0.08 | -0.12* | 0.104* | 0.16** | -0.06 | 0.32** | -0.11* |  | 0.31** | 0.03 | -0.07 | 0.03 | 0.11* | 0.08 | 0.09 | 0.03 | -0.17** | 0.10* | 0.13** |
| HFC | 0.03 | 0.06 | -0.07 | 0.00 | -0.10 | -0.05 | -0.02 | -0.05 | 0.03 | -0.12* | -0.03 | 0.16** | 0.03 | 0.11* | 0.00 | 0.24** |  | 0.02 | 0.00 | -0.13** | -0.12* | -0.03 | -0.09 | -0.03 | -0.09 | 0.04 | -0.01 |
| FS | 0.15** | 0.07 | -0.01 | -0.01 | -0.07 | -0.14** | -0.01 | 0.01 | -0.04 | -0.12* | 0.04 | 0.11* | -0.02 | 0.09 | 0.05 | 0.07 | 0.06 |  | -0.23** | 0.14** | 0.13** | 0.09 | 0.06 | 0.47** | -0.05 | -0.06 | 0.04 |
| FC | -0.10 | -0.08 | 0.01 | 0.01 | 0.03 | 0.25** | 0.20** | 0.15** | 0.00 | 0.23** | -0.06 | -0.12* | 0.02 | -0.15** | 0.00 | -0.10 | 0.07 | -0.23** |  | -0.19** | -0.19** | -0.19** | -0.34** | -0.16** | 0.34** | -0.02 | -0.30** |
| EF | -0.15** | -0.14** | 0.17** | 0.13* | 0.12* | 0.08 | -0.05 | 0.10 | 0.01 | 0.03 | 0.15** | -0.03 | 0.00 | -0.05 | 0.20** | 0.13* | 0.04 | 0.11* | -0.05 |  | 0.57** | 0.04 | 0.24** | 0.04 | -0.03 | -0.17** | -0.01 |
| DF | -0.12* | -0.04 | 0.02 | -0.04 | 0.04 | -0.04 | -0.10* | 0.03 | -0.02 | -0.05 | 0.15** | 0.09 | -0.04 | 0.00 | 0.13* | 0.14** | -0.01 | 0.14** | -0.15** | 0.67** |  | 0.07 | 0.21** | 0.08 | -0.11* | -0.05 | 0.09 |
| FF | 0.09 | 0.13** | -0.04 | -0.01 | -0.04 | -0.05 | -0.04 | 0.06 | 0.02 | -0.26** | -0.06 | 0.08 | 0.04 | 0.07 | -0.01 | 0.07 | 0.06 | 0.09 | -0.12* | 0.12* | 0.17** |  | 0.34** | 0.07 | -0.13** | 0.03 | 0.16** |
| MF | 0.20** | 0.26** | -0.04 | -0.02 | -0.01 | -0.01 | 0.02 | 0.06 | 0.00 | -0.25** | -0.02 | 0.14** | -0.08 | 0.18** | -0.01 | 0.14** | -0.01 | 0.13** | -0.19** | 0.13* | 0.18** | 0.45** |  | -0.02 | -0.06 | 0.01 | -0.02 |
| FAMS | 0.22** | 0.14** | -0.13* | -0.05 | -0.15** | -0.20** | -0.02 | 0.00 | -0.02 | -0.13** | -0.03 | 0.18** | 0.06 | 0.05 | -0.03 | 0.06 | 0.03 | 0.44** | -0.15** | 0.07 | 0.15** | 0.10* | 0.20** |  | -0.35** | -0.07 | 0.14** |
| FAMC | -0.07 | -0.11* | 0.11* | 0.06 | 0.12* | 0.32** | 0.16** | 0.29** | -0.04 | 0.29** | -0.09 | -0.17** | 0.00 | -0.13** | 0.03 | -0.11* | -0.04 | -0.09 | 0.38** | -0.01 | -0.16** | -0.06 | -0.06 | -0.24** |  | -0.03 | -0.47** |
| FIH | 0.10* | 0.20** | -0.12* | -0.11* | -0.14** | -0.09 | 0.05 | 0.01 | -0.07 | -0.10* | -0.11* | 0.17** | -0.04 | 0.13** | 0.01 | 0.02 | 0.01 | 0.06 | -0.04 | -0.05 | -0.02 | 0.12* | 0.16** | 0.02 | 0.05 |  | 0.13* |
| HSL | 0.14** | 0.10* | -0.27** | -0.20** | -0.28** | -0.43** | -0.32** | -0.32** | -0.01 | -0.39** | 0.02 | 0.46** | -0.07 | 0.40** | -0.02 | 0.22** | 0.06 | 0.22** | -0.31** | 0.02 | 0.11* | 0.09 | 0.12* | 0.19** | -0.25** | 0.12* |  |

* *p* <0.05; ** *p* <0.001
